# The Algorithmic Lung Detective: Artificial Intelligence in the Diagnosis of Pulmonary Embolism

**DOI:** 10.7759/cureus.51006

**Published:** 2023-12-23

**Authors:** Nishant Allena, Sneha Khanal

**Affiliations:** 1 Pulmonary Medicine, BronxCare Health System, Bronx, USA; 2 Internal Medicine, BronxCare Health System, Bronx, USA

**Keywords:** artificial intelligence ct, artificial intelligence in pulmonology, ai and machine learning, pulmonary embolism (pe), artificial intelligence in medicine

## Abstract

Pulmonary embolism (PE) poses a significant threat as the third leading cause of cardiovascular death, prompting the widespread use of CT pulmonary angiogram for rapid detection. Despite its prevalence, diagnostic accuracy remains variable among radiologists. The emergence of artificial intelligence (AI), notably through convolutional neural networks and deep learning reconstruction, offers a promising avenue to enhance PE detection. AI demonstrates superior sensitivity and negative predictive values, reducing the risk of missed diagnoses. Implementation of AI-based worklist prioritization substantially shortens detection and notification times, streamlining radiological workflows. However, it is crucial to underscore that AI acts as a complement, not a replacement, for radiologists, synergizing with human expertise. As AI integration progresses, it holds the potential to significantly improve diagnostic accuracy and efficiency in pulmonary embolism detection while maintaining the essential role of human judgment in medical decision-making.

## Editorial

Pulmonary embolism stands as the third most common cause of cardiovascular death and is also a significant cause of in-hospital mortality [1]. The incidence of venous thromboembolism, including both pulmonary embolism (PE) and deep vein thrombosis has been noted to be approximately 100 to 150 per 100,000 people [2,3]. The gold standard investigation used to aid in the rapid detection of pulmonary embolism is a CT pulmonary angiogram with a sensitivity of 83% and specificity of 96% [4], which is performed in a majority of emergency departments and hospitals around the world.

Various studies have been done to assess the accuracy in the diagnosis of PE and the question about interobserver bias in the diagnosis of PE has remained in discussion over the years. Studies done to assess the variability in the diagnosis of PE among radiologists with different levels of experience showed that four out of five radiologists with experience of two years or more missed the peripheral emboli in 4-6/290 patients [5]. The rate of missed incidental pulmonary embolism is variable but has been noted to be as high as 44.8%, however, the missed rate of pulmonary embolism went down to 2.6% with the use of AI assistance [6]. Studies have demonstrated the missed diagnosis of pulmonary embolism, later detected by AI on retrospective analysis to be 38% [7]. Historically, the mortality and recurrence rates for untreated or missed pulmonary embolisms were quoted to be a whopping high of 26-30%, with a decline in the rates noted to be 5% in ambulatory settings [8].

AI's journey in medical imaging began in the 1960s but was hindered by technological constraints likely due to the limited amount of digital data available at the time to train algorithms and the lack of powerful computational hardware. The widespread integration of AI in radiology reporting, marked by the introduction of artificial neural networks and computer-aided detection software in the 1980s, has enabled advancements such as routine use in mammography, where it consistently improves lesion detection with minor increases in recall rates [9]. The current AI systems utilized in CT scans and MR interpretations are starting to gain popularity in the radiology world. The convolutional neural network (CNN)-based deep learning approach has notably been the most popular AI tool used to reduce image noise, develop contrast modality images for image segmentations, harmonization, and even synthesis of the missing modality popularly called deep learning reconstruction (DLR) [10,11]. Other algorithms for deep learning methods include random forests (RF) and support vector machines (SVM) among others for DLR and image post-processing [12].

A study showed that when compared to clinical reports, AI showed a notably lower positive predictive value (PPV) (86.8% compared to 97.3%, with a p-value of 0.03) and slightly lower specificity (99.8% compared to 100%, with a p-value of 0.045), without any significant differences in sensitivity and negative predictive value (NPV) in the detection of incidental PEs [13]. In a study conducted by Cheik et al., software utilizing artificial intelligence (AI) for image interpretation demonstrated the ability to identify 219 suspicious cases of pulmonary embolisms (PEs), of which 176 were confirmed as true PEs. Notably, 19 of these true PEs were initially missed by radiologists. The AI system exhibited the highest sensitivity and negative predictive values (NPVs) at 92.6% and 98.6%, respectively, surpassing the radiologists who achieved 90% sensitivity and a 98.1% NPV. On the other hand, radiologists excelled in terms of specificity and positive predictive value (PPV), with 99.1% specificity and a 95% PPV compared to the AI's 95.8% specificity and 80.4% PPV. These results highlight the potential of AI to enhance PE detection, particularly in identifying true positives, while radiologists excel in reducing false positives [14].

The implementation of AI-based worklist prioritization resulted in a substantial reduction in the median turnaround time (TAT) from 7772 minutes for examinations showing evidence of pulmonary embolism (IPE) to just 148 minutes [6]. Radiology has played a pioneering role in introducing AI into the field of medicine. Based on observations similar to the ones mentioned above, it is reasonable to anticipate a substantial replacement of radiologists by AI or at the very least incorporation of AI into the core radiology curriculum to enhance productivity as a reality. By that time, we can presume that the integration of AI in various medical and non-medical domains will also have made significant progress.

The ethical application of AI in radiology mandates a commitment to enhancing well-being, minimizing harm, and ensuring fair distribution of benefits and risks among stakeholders. Critical elements include transparency and reliability, aiming to eliminate bias in decision-making while upholding human accountability in both AI design and operation. The radiology community is encouraged to proactively establish ethical codes and guidelines for AI implementation. Despite AI's growing role, radiologists remain ultimately responsible for patient care, necessitating the acquisition of new skills to navigate the evolving AI landscape. This ethical framework seeks to harmonize technological progress with human-centered values, emphasizing a just and accountable approach to integrating AI into radiology practices [15].

Based on our comprehensive analysis, we posit that the incorporation of AI into the pulmonary embolism detection process serves a twofold purpose. First, it functions as a crucial safety net, substantially diminishing the probability of missing pulmonary embolisms. Second, it aids radiologists in expediting their reporting process. It is imperative to underscore that AI does not supplant the role of the radiologist, as human judgment and expertise remain indispensable for making critical decisions, particularly in light of the inherent biological variability inherent in the human body.
